# Is Payoff Necessarily Weighted by Probability When Making a Risky Choice? Evidence from Functional Connectivity Analysis

**DOI:** 10.1371/journal.pone.0041048

**Published:** 2012-07-17

**Authors:** Li-Lin Rao, Shu Li, Tianzi Jiang, Yuan Zhou

**Affiliations:** 1 Key Laboratory of Behavioral Science, Magnetic Resonance Imaging Research Center, Institute of Psychology, Chinese Academy of Sciences, Beijing, China; 2 LIAMA Center for Computational Medicine, National Laboratory of Pattern Recognition, Institute of Automation, Chinese Academy of Sciences, Beijing, China; 3 Key Laboratory for NeuroInformation of Ministry of Education, School of Life Science and Technology, University of Electronic Science and Technology of China, Chengdu, China; 4 The Queensland Brain Institute, The University of Queensland, Brisbane, Queensland, Australia; University of Maryland, College Park, United States of America

## Abstract

How people make decisions under risk remains an as-yet-unresolved but fundamental question. Mainstream theories about risky decision making assume that the core processes involved in reaching a risky decision include weighting each payoff or reward magnitude by its probability and then summing the outcomes. However, recently developed theories question whether payoffs are necessarily weighted by probability when making a risky choice. Using functional connectivity analysis, we aimed to provide neural evidence to answer whether this key assumption of computing expectations holds when making a risky choice. We contrasted a trade-off instruction choice that required participants to integrate probability and payoff information with a preferential choice that did not. Based on the functional connectivity patterns between regions in which activity was detected during both of the decision-making tasks, we classified the regions into two networks. One network includes primarily the left and right lateral prefrontal cortices and posterior parietal cortices, which were found to be related to probability in previous reports, and the other network is composed of the bilateral basal ganglia, which have been implicated in payoff. We also found that connectivity between the payoff network and some regions in the probability network (including the left lateral prefrontal cortices and bilateral inferior parietal lobes) were stronger during the trade-off instruction choice task than during the preferential choice task. This indicates that the functional integration between the probability and payoff networks during preferential choice was not as strong as the integration during trade-off instruction choice. Our results provide neural evidence that the weighting process uniformly predicted by the mainstream theory is unnecessary during preferential choice. Thus, our functional integration findings can provide a new direction for the investigation of the principles of risky decision making.

## Introduction

Life is full of changes and uncertainties, and people continually make day-to-day decisions in the presence of uncertainty. A decision involves risk if the decision maker does not know which states of nature will occur but does know their probabilities of occurring. A decision can be considered to involve uncertainty if the decision maker has no information about the relative likelihood of the various states of nature (for a more detailed distinction see [1]).

The first normative theory applied to decision making under risk was the Expected Value (EV) theory, which was developed by Blaise Pascal and Pierre de Fermât in the 1600s. Numerous models based on this theory have been proposed to describe decision making under risk. According to Lopes [2], two fundamentally different mechanisms have been proposed to explain decisions making under risk according to the EV theory. One proposed mechanism is the family of weighted utility models that originated with Bernoulli [3], and the other is the family of rank-dependent value models independently proposed by a number of authors [4]–[8].

Taking a closer look at these two families reveals that they have one thing in common: both model families assume that risky decisions are made by choosing the option that offers the greatest expected value or utility which is the sum of all potential payoffs *u*(*x*) weighted by their corresponding (transformed) probabilities *f*(*p*), 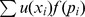. That is, decision-makers are required to 1) compute the mathematical expectation of each option by weighting and summing and then 2) choose the option that maximizes the overall expected value or utility.

Although many decision models, which are assumed to differ from each other, have been formed, only one valuation principle–the expectation principle–exists, and it is relatively under-studied [9]. A desire to justify the validity of this valuation rule has long motivated the creation of generalized alternatives [5], [10]–[15]. That is, numerous theories have been conceived in an attempt to demonstrate that, with an appropriate transformation of outcomes or an appropriate transformation of the outcome probabilities, this expectation principle will satisfactorily explain the data. The main body of research related to the analysis of choice under risk is built upon just one theory, the EV theory, and almost all existing psychological models revolve around only one decision rule. However, the expectation principle (i.e., expectation maximization or expectation minimization), which leads to the general model of expectation, is open to criticism.

It is not clear that the weighting process actually represents what happens when people make a risky decision. The processes of weighting and summing have long been challenged (e.g., ([16]–[18]), and some behavioral studies have provided experimental evidence that decision making does not involve a weighting process during preferential choice (for overviews see [19], [20]). However, behavioral observations are not able to directly examine the internal weighting process that underlies decision making under risk, and thus the debate on the weighting process remains unresolved. Functional connectivity analysis, which measures the temporal correlations of blood-oxygen-level-dependent (BOLD) signals obtained by functional magnetic resonance imaging, provides an approach to examine the neural activity underlying the prescribed pattern in a risky decision and thus has the potential to provide neural evidence for the weighting process debate. Functional connectivity analysis can be used to identify the organization, interrelationships and integrated performance of different regions of the brain. This approach has been used to elucidate the ways that different brain regions interact during decision making [21]–[23]. The strength of functional connectivity is thought to reflect the extent of coordination between brain regions [24], [25]. In the present study, we used functional connectivity analyses to re-analyze previously published data [26] to answer the question of whether payoffs are necessarily weighted by probability when making a risky choice.

Neuroeconomic studies have attempted to identify the neural substrates associated with probability and payoff. The posterior parietal cortex (PPC) and the lateral prefrontal cortex (PFC) have been implicated as the core regions in the probability network involved in the decision-making process. The PPC, because of its role in numerical estimation and calculation [27], is believed to be involved in the representation of probability [28]–[30]. The lateral PFC, which is generally accepted to participate in cognitive control [31] and information updating and maintenance [32], [33], may also play a vital role in processing probability information during a conscious deliberation about a pair of risky options. Recent studies have further demonstrated the presence of nonlinear probability weighting functions in a network of areas that include the PPC [34] and the lateral PFC [35]. Evidence about the neural substrates of uncertainty also implicates these regions in probability information processing [36]–[38]. However, the neural substrate most consistently reported as being sensitive to outcome magnitude is the striatum, including the dorsal and ventral striatum (and especially the nucleus accumbens). Although some investigators linked the striatum with expected value [39], the striatum, which is commonly considered to be the core of the reward circuit [40]–[42], has repeatedly been found to be correlated with the magnitude of anticipated and experienced rewards during decision making [38], [43]–[46].

We hypothesized that if a payoff must necessarily be weighted by its probability when making a risky choice, then the coordination between the brain activity associated with probability representation and that associated with payoff representation should be reasonably strong. This tight coordination would be revealed by strong functional connectivity between regions associated with probability representation and those associated with payoff representation. Alternatively, if a payoff need not be weighted by its probability, suggesting that a decision is made based on a single dimension (payoff or probability) [9], [17], [19], [47], [48], then the regions associated with probability representation would not necessarily be coordinated with those associated with payoff representation. Thus, the functional connectivity between regions associated with probability representation and those associated with payoff representation would not be expected to be particularly strong when making a risky choice. To test our hypothesis, we used functional connectivity analyses to re-analyze previously published data [26] in which a trade-off instruction choice task and a preferential choice task were used. Although the trade-off instruction choice task required that the participants integrate information from the probability and payoff dimensions [49], [50], the preferential choice task did not. We predicted that if the payoffs were necessarily weighted by their respective probabilities during a preferential choice, we would find no difference in the functional connectivity between regions associated with probability representation and those associated with payoff representation between the preferential choice and the trade-off instruction choice.

## Materials and Methods

### Ethics Statement

This study was approved by both the Institutional Review Board of the Institute of Psychology, Chinese Academy of Sciences, and the Institutional Review Board of the Beijing MRI Center for Brain Research. All participants gave written informed consent.

### Participants

Twenty-nine undergraduates or postgraduates were recruited to participate in this study. Three participants finished only one task (the preferential choice task or the trade-off instruction task) and thus were excluded in our data analyses. Among the remaining 23 participants (10 males, mean age 22 years, SD 2.82) whose data were utilized in this study, three participants were excluded from all analyses because of excessive head motion. Furthermore, two participants who exhibited short and sudden head motion in the first trial were excluded from the functional connectivity analyses (for details, please see the fMRI preprocessing section). All participants were in good health with no previous history of psychiatric or neurological disease.

### Task Description

Stimuli were presented with E-prime software (Psychology Software Tools, Pittsburgh, PA) on a personal computer and were back-projected onto a screen using a liquid crystal display projector and viewed by participants through a mirror mounted on the MRI head coil. All participants were presented with a total of 60 pairs of two-outcome monetary bets. Each pair of bets was comprised of one bet featuring a high probability (81% to 97%) of winning/losing a modest sum of money (the *P* bet) and another featuring a low probability (19% to 39%) of winning/losing a comparatively large amount of money (the *$* bet). The expected values for the *P* bets and the *$* bets ranged from ±16 to ±44 Chinese Yuan (CNY).

**Figure 1:**
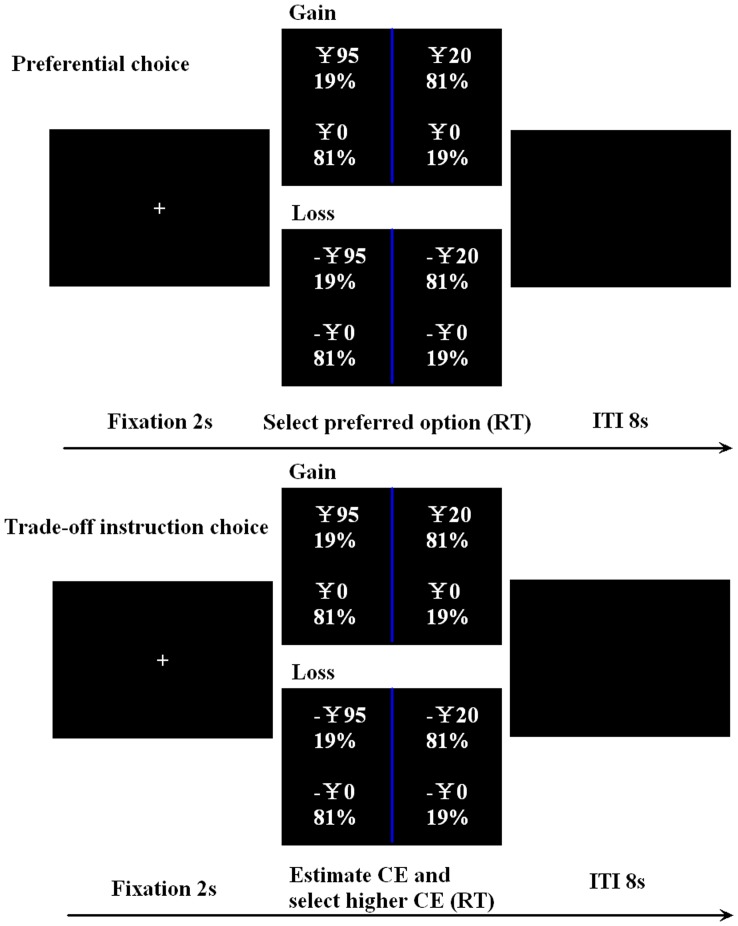
Experimental design. CE denotes certainty equivalence.

Figure 1 illustrates the experimental design. In the preferential choice task, the participants were asked to select their preferred option from each pair. Considering that the certainty equivalent method was based on a compensatory rule [50], in the trade-off instruction choice task, the participants were asked to perform a compensatory process of trading off probability against payoff by using the certainty equivalent method [51], [52]. In particular, the participants were asked to estimate the amount of cash equivalent that would make them indifferent to each bet and select the bet with the higher certainty equivalence. The tasks were the same as those used in our previous study (Rao, Zhou et al. 2011). The order of the tasks was counterbalanced between the participants, with an interval of at least 7 days. The order of the trials within each domain was randomized, and the order of the domain was counterbalanced between the two tasks and within the participants.

Before scanning, the participants were instructed in the task and tested for task comprehension. During the scanning session, each participant completed 60 trials of each task. Each trial began with a 2-s fixation cross, followed by one pair of bets. Participants were instructed to make their decisions by pressing one of the two buttons corresponding to the location of the options on the screen, with no time constraints. Following the button press, there was a delay of 8 s (Figure 1). At the end of each task, two of the participants’ decisions were randomly selected to determine their payoff (one in the gain domain and the other in the loss domain). At the completion of the study, the participants were paid ¥100 in cash for participating, and the losses or winnings determined by the above method were deducted from or added to the final payment.

### fMRI Scanning Procedure

Images were acquired with a 3.0 Tesla Siemens MRI scanner. Whole-brain functional scans were collected in 32 axial slices using an echo-planar imaging (EPI) sequence (repetition time = 2000 ms echo time = 30 ms; flip angle = 90°, matrix = 64×64; field of view = 220×220 mm^2^; slice thickness = 3 mm; slice gap = 1 mm, final acquisition voxel size = 3.4×3.4×4 mm^3^).

### fMRI Preprocessing

Image preprocessing was performed using statistical parametric mapping (SPM5, Wellcome Department, London, UK) running on a Matlab 7 platform (MathWorks, Natick, MA). The preprocessing included slice time correction, realignment, normalization and resampling to 3×3×3 mm^3^, and smoothing using an 8-mm full-width-at-half-maximum Gaussian kernel. Based on their recorded motion correction estimates, three subjects who had more than a 2-mm maximum displacement in any of the x, y or z directions or more than 2° of angular rotation about any axis for multiple volumes were excluded from this study. Two additional participants exhibited short and sudden head motion during the first trial. The data obtained from these two subjects were used in activation detection after removing the first trial, but were excluded from the functional connectivity analyses.

For the data used in connectivity analyses, the temporal autocorrelation associated with linear trends due to session-related signal variance, movement related artifacts, and physiological artifacts (including mean global signal, white matter and cerebrospinal fluid signal) was removed through linear regression. Next, a temporal bandpass filter (0.01–0.1 Hz) was used to preserve the BOLD while removing higher- and lower-frequency noise. These procedures are similar to previous studies [22], [53], [54].

### Functional Connectivity Analyses and Statistics

To test our hypothesis about whether the functional connectivity of regions associated with probability representation and payoff representation during the preferential choice task were different from those associated with the trade-off instruction choice task, functional connectivity analyses were performed. Because brain regions do not act in isolation from each other but rather must work together as a system [22], we first investigated whether the regions participating in the decision-making tasks could be categorized into two networks. These include a probability network composed of regions associated with probability representation and a payoff network composed of regions associated with payoff representation based on their interregional functional connectivity pattern. We then assessed differences in the functional connectivity patterns across conditions by investigating the functional connectivity between the two networks and investigating the functional connectivity of each network using a voxel-by-voxel approach.

#### Activation detection and selection of seed regions

The seed regions were selected from the regions that showed activation in all four task conditions. The methods of activation detection were the same as that in our previous paper, which focused on differences in activation between preferential choice and trade-off instruction choice in conflict-related regions [26]. However, in this study, we attempted to detect all regions activated in each task condition (i.e., the gain and loss domains in both the preferential and trade-off instruction choices). Briefly, general linear model analyses were used to detect the brain activity of each participant during the decision epochs for each task condition. Events were modeled with a variable-duration boxcar function convolved with a canonical hemodynamic response function. For subject-level analysis, images of the parameter estimates for the contrast of interest (task > baseline) were created for each participant to identify the regions that showed relatively high activity during the decision epochs (activated regions) for each task condition. These contrast images were tested with one-sample t-tests to permit inferences at the group level (i.e., a second-level analysis) (p<0.01, corrected by FDR). Conjunction analysis was then used to identify the regions commonly activated across the four conditions. To make the conjunction analysis at the second level executable in SPM5, a one-way ANOVA was performed, enabling the contrast images (task > baseline) for each task obtained at the first level to be entered into the model at the same time. After estimating the model, we specified the contrasts for each task condition and then selected all contrasts together using ctrl+click to perform the conjunction analysis at the second level. A strict threshold was selected to obtain cortical regions that were activated in all four tasks (p<0.0001, corrected by FDR). This threshold ensured that the coordinates for the independent peaks that represent different functional regions within a larger cluster could be obtained while still enabling the detection of activation in the cortex. For the subcortical regions, a looser threshold (p<0.01, corrected by FDR) was used to obtain the bilateral activation regions in the basal ganglia. A number of studies have suggested that discrete regions of the striatum contribute differently to decision-making through functional integration with regions involved with processing sensorimotor, cognitive, and motivational/emotional information [55]. Therefore, the striatum must be further partitioned into its dorsal and ventral areas. To obtain seed regions that could be isolated to the dorsal or ventral striatum, several steps were performed. First, we extracted the bilateral caudate and putamen template regions using a prior anatomical automatic labeling (AAL) atlas [56], with the WFU_pickatlas tool (http://fmri.wfubmc.edu/software/PickAtlas) Next, the caudate and putamen template regions were divided into dorsal and ventral areas, defined as z >2 (dorsal) or z ≤2 (ventral) for the putamen and z >7 (dorsal) or z ≤7 (ventral) for the caudate; these values were chosen based on a meta-analysis [57]. The dorsal and ventral striatum template regions thus generated were compared to our activation map to obtain the peak coordinates in the dorsal and ventral striatum, respectively (p<0.01, corrected by FDR). In a similar manner, we generated the peak coordinates in the bilateral globus pallidus and the thalamus.

Finally, the seed regions were identified as the intersection between the activated clusters obtained by the conjunction analysis and the 18-mm (for the cortical regions) or 12-mm (for the subcortical regions) diameter spheres centered on these peak coordinates. The selection of the diameter of the spheres was similar to that used in previous studies [57]–[59].

#### Identification of the interregional functional connectivity patterns within each condition

To inspect the interregional functional connectivity patterns, the mean time series of each seed region was acquired by averaging the time series of all voxels within that region. The 6 TR intervals following the decision cue were marked to isolate those time points. The time course of each seed region was then spliced and concatenated to include only data from the decision epochs. This process of splicing and concatenating data has been used in previous studies on decision-making [21]–[23]. Next, Pearson’s correlation coefficients were computed between each pair of these regions. Thus, for each participant, we obtained a connectivity matrix, with each element representing the strength of functional connectivity between two corresponding seed regions. After the correlation coefficients were converted to z values using Fisher’s r-to-z transform to improve their normality, two-tailed, one-sample t-tests were performed for all possible pairwise correlations across subjects to determine whether each interregional correlation significantly differed from zero for each condition (p<0.05, corrected by FDR).

To investigate whether potential functional modules (networks) encoded in the network topology could be uncovered automatically, an average-linkage hierarchical clustering algorithm was used to analyze the averaged interregional functional connectivity matrix within each condition. Applying the hierarchical clustering algorithm, which was implemented using Matlab, to the connectivity matrix identified those regions that have a high similarity to each other. Therefore, with hierarchical clustering analyses, we were able to divide these seed regions into different networks [60].

#### Detecting differences in the functional connectivity of the regions associated with probability and payoff

To detect differences in the functional connectivity patterns across conditions, two types of connectivity analyses were undertaken.

- Functional connectivity analysis between networks. Based on the interregional functional connectivity patterns within each condition revealed by the above analyses, the seed regions could be grouped into different networks (two networks were found in this study). The mean time series of each seed-network region was acquired by averaging the time series of all voxels within that network. After performing Fisher’s r-to-z transform, Pearson’s correlation coefficients were computed between the mean time series of every pair of seed-network regions. A two-way, within-subject, repeated-measures ANOVA (RMANOVA) involving a 2 (task: preferential choice vs. trade-off instruction choice) by 2 (domain: gain vs. loss) design was performed (p<0.05).
- A voxel-wise functional connectivity analysis based on each individual network. In this analysis, the networks themselves were selected as the new seed regions (seed networks) for the voxel-wise functional connectivity analysis. For each seed-network region, correlation maps were produced by computing the Pearson’s correlation coefficients between the mean time series of this seed-network region and the time series for each voxel in the brain. After applying Fisher’s r-to-z transform, the z-values for each individual in the correlation map were entered into a second-level analysis in SPM. RMANOVAs were performed to detect the functional connectivity identified by the main effect of task or domain and the interaction effect (p<0.05, corrected by FDR, with a cluster threshold of 6 contiguous voxels). To restrict our analysis, masks were used. While analyzing the functional connectivity of one seed-network region, the seed regions that constituted the other network were combined as the mask.

Finally, to clarify the possibility that the difficulty of the task indicated by the activation extent may influence the strength of functional connectivity, a RMANOVA was performed to detect the main effect of task or domain and the interaction effect on the activation extent of brain regions that were selected to conduct the functional connectivity analysis. As we focused on the regions that were selected to conduct the functional connectivity analysis, a small volume correction was used on the mask composed of these seed regions (FWE-corrected p<0.05).

## Results

### Common Neural Basis for the Preferential and Trade-off Instruction Choices

**Figure 2:**
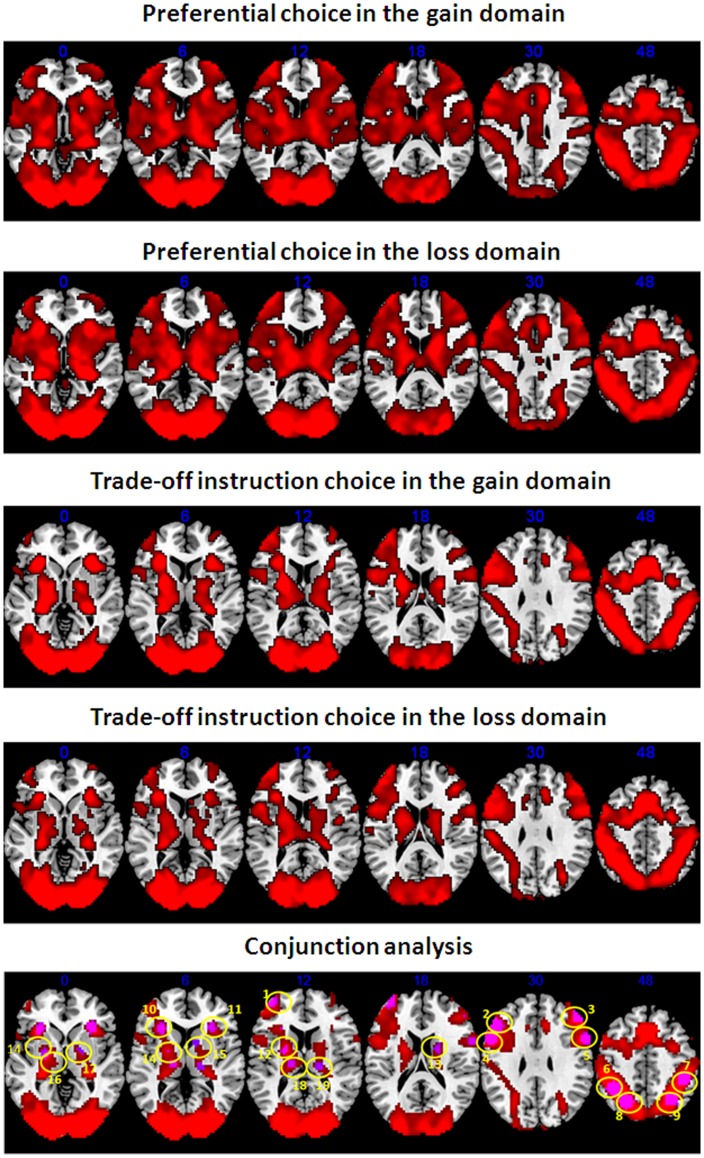
Activated regions across the four conditions obtained by conjunction analysis. The activated regions are shown in red. For the presentation purposes, a threshold of p<0.01 (corrected by FDR) was used. The seed regions for functional connectivity are shown in purple. Please see Table 1 for a detailed interpretation of the numbers.

In the preferential and trade-off instruction choice tasks, the whole brain analyses identified regions with significant activity across all decision epochs. Conjunction analyses showed that the bilateral lateral prefrontal cortices, posterior parietal cortices, basal ganglia areas and motor and visual cortices were commonly activated across the two tasks in both the gain and the loss domains (p<0.01, corrected by FDR) (Figure 2). Combining the activation patterns we detected with the results from prior literature, the bilateral frontal regions (including the left anterior prefrontal cortex, bilateral middle frontal cortices and inferior frontal cortices) [36]–[38], the bilateral posterior parietal regions (including the superior parietal lobes and inferior parietal lobes) [28]–[30], [34], [36], [61], the bilateral anterior insula [36], [62], [63] and the bilateral basal ganglia (including the dorsal and ventral striatum, pallidum and thalamus) [38], [43]–[45] were selected as the seed regions for subsequent functional analyses (Table 1). The clusters in the visual and motor cortices were excluded from the seed regions because the visual and motor cortices are unrelated to uncertainty [36] and were probably activated by the need to process and respond to the task state. The bilateral nucleus accumbens and medial prefrontal cortices (two regions implicated in risky decision making in previous literature, e.g., [43], [64]) were not included in our analyses because these regions were not detected by the conjunction analysis (even at a threshold of p<0.01, uncorrected). In total, 19 seed regions were selected.

**Table 1:** Seed regions for the interregional functional connectivity analyses.

NO.	Seed region	BA	Coordinate (x,y,z)
1	L.aPFC	10	−39,57,18
2	L.MFG	46	−48,27,30
3	R.MFG	46	51,39,27
4	L.MFG/IFG(pIFS)	9	−54,12,36
5	R.MFG/IFG(pIFS)	9	60,12,24
6	L.SPL	7	−24, −63,48
7	R.SPL	7	30, −60,51
8	L.IPL	40	−39, −48,48
9	R.IPL	40	45, −36,45
10	L.aINS	13	−27,24,3
11	R.aINS	13	30,27,0
12	L.dSTR*		−21,3,12
13	R.dSTR*		21,0,18
14	L.vSTR*		−24, −6,0
15	R.vSTR*		12,6,6
16	L.PAL*		−21, −9,0
17	R.PAL*		18,−3,−3
18	L.THA*		−15, −18,9
19	R.THA*		18, −21,9

* Seed regions were generated from 12-mm diameter spheres centered on the peak foci of the region. The other seed regions were generated from 18-mm diameter spheres centered on the peak foci. For details, please see the main text. Abbreviations: L: left; R: right; aPFC: anterior prefrontal cortex; aINS: anterior insula; BA: Brodmann area; dSTR: dorsal striatum; IFG: inferior frontal cortex; IPL: inferior parietal lobe; MFG: middle frontal cortex; PAL: pallidum; pIFS: posterior inferior frontal sulcus; SPL: superior parietal lobe; THA: thalamus; vSTR: ventral striatum.

### Interregional Functional Connectivity Patterns within Each Task Condition

**Figure 3:**
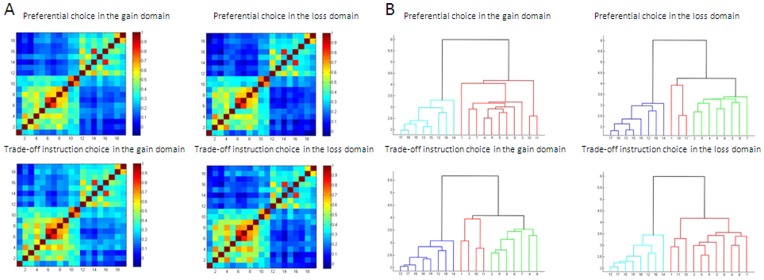
The mean r-value matrices (A) and hierarchical clustering analyses (B) for the four task conditions: the preferential choice in the gain domain, the preferential choice in the loss domain, the trade-off instruction choice in the gain domain and the trade-off instruction choice in the loss domain. (A): Each figure shows a 19×19 square matrix in which the x and y axes correspond to the regions listed in Table 1 and in which each entry indicates the mean strength of the functional connectivity between each pair of brain regions. The z score of the functional connectivity is indicated with a colored bar. (B): The vertical axis represents distance. The numbers in the horizontal axis represent the seed regions. Please see Table 1 for a detailed interpretation of the numbers.

The connectivity matrix within each task condition showed that the interregional functional connectivity patterns were similar across the four task conditions, but with differences in connectivity strength (Figure 3). The connectivity matrix within each task condition revealed that the seed regions could be clearly divided into two networks. One of these was composed of the bilateral prefrontal regions, parietal regions and insula, all of which have been demonstrated in previous studies to participate in risk or probability information processing (for details, please see the discussion). Therefore, we named this network the probability network. The other network was composed of the subcortical regions, including the bilateral striatum and thalamus, which have been linked to reward magnitude in previous studies (for details, please see the discussion), and thus we called this the payoff network.

### Differences in Functional Connectivity between Regions Associated with Probability and Payoff

#### Functional connectivity analysis between networks

We conducted a 2 (task: preferential choice vs. trade-off instruction choice) ×2 (domain: gain vs. loss) RMANOVA to compare differences in the strength of functional connectivity between the seed-network regions. The results revealed marginally significant main effects of task (F(1, 20) = 3.26, p = 0.086.), with stronger connectivity found in the trade-off instruction task (mean = 0.35, std = 0.23) than in the preferential choice task (mean = 0.29, std = 0.22). We found no main effects of domain (F(1, 20) = 0.41, n.s.) or of the interaction between task and domain (F(1, 20) = 0.004, n.s).

#### A voxel-wise functional connectivity analysis based on individual networks

**Figure 4:**
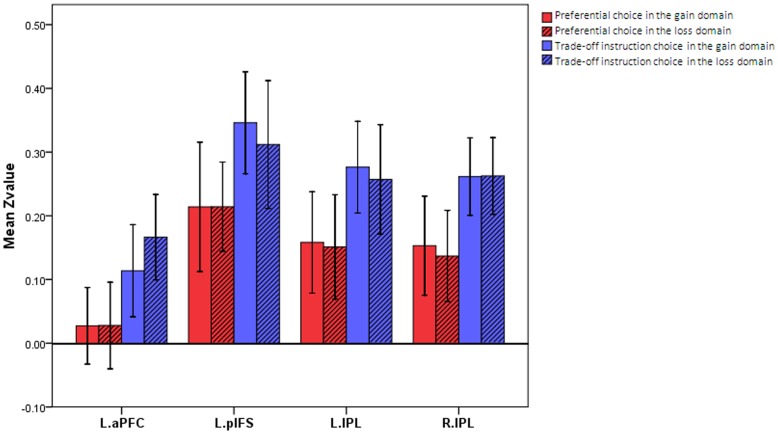
Mean correlation coefficients in the regions in the probability network showing differences in connectivity with the payoff network. Abbreviations: see Table 1.

While taking the payoff network as the seed region for a voxel-wise functional connectivity analysis, we found a significant main effect of task in the connectivity between the payoff network and the regions within the probability network, including the left lateral prefrontal regions and the bilateral inferior parietal lobes (p<0.05, corrected by FDR). Further analyses revealed that all of these connectivities were stronger during the trade-off instruction task than during the preferential choice task. No main effects of domain or interaction were found (Table 2, Figure 4).

**Table 2:** Differences in functional connectivity associated with the payoff network or the probability network (p<0.05, corrected by FDR).

Seed_network_	Connected region	BA	Coordinate (x,y,z)	PCG	PCL	TCG	TCL	F value	P_FDR-corr_ value
				z value:mean/std	z value:mean/std	z value:mean/std	z value:mean/std		
**Main effect of condition**									
payoff	L.aPFC	10	−42,54,15	0.03/0.14	0.03/0.16	0.11/0.17	0.17/0.15	20.78	0.01
	L.pIFS	9	−51,9,36	0.21/0.23	0.21/0.16	0.35/0.18	0.31/0.23	8.95	0.04
	L.IPL	40	−36,−48,42	0.16/0.18	0.16/0.19	0.28/0.16	0.26/0.2	14.63	0.014
	R.IPL	40	45,−42,51	0.15/0.18	0.14/0.16	0.26/0.14	0.26/0.14	20.65	0.01
probability	none	−							
**Main effect of domain**									
payoff	none								
probability	none								
**Interaction effect**									
payoff	none								
probability	none								

Abbreviations: PCG: the preferential choice in the gain domain; PCL: the preferential choice in the loss domain; TCG: the trade-off instruction choice in the gain domain; TCL: the trade-off instruction choice in the loss domain. L: left; R: right; aPFC: anterior prefrontal cortex; IPL: inferior parietal lobe; pIFS: posterior inferior frontal sulcus.

When we took the probability network as the seed region, we found no significant main effect of task, domain or interaction (p<0.05, corrected by FDR).

### Differences in the Activation Extent in Seed Regions of Functional Connectivity

**Figure 5:**
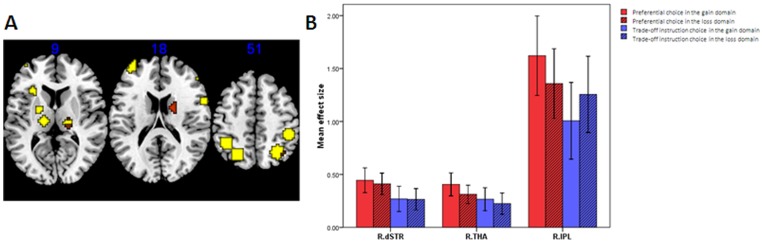
Differences in the activation extent in the seed regions of the functional connectivities. (A) Clusters showing the differences in activation extent are in red, and the seed regions for the functional connectivities are in yellow. (B) The parametric estimates of these clusters showing the differences in activation extent during each task condition are also shown. Abbreviations: see Table 1.

The RMANOVA showed that the significant main effect of task was found in the right dSTR (MNI coordinate of peak voxle: [15,0,18]), the right THA (MNI coordinate of peak voxle:[18,−27,9]), and the right IPL (MNI coordinate of peak voxle:[39,−60,51]) (FWE-corrected p<0.05). Taking a closer look to this effect, we found that all these clusters showed stronger activation in the preferential choice than in the trade-off instruction choice (Figure 5). No other effects were found.

## Discussion

A large body of behavioral literature suggests that a decision between simple gambles can be fitted by an expectation computation model such as the Expected Utility or the Cumulative Prospect Theory [5], [13], [15], [65]. Although this view has long been challenged (e.g., [16], [17], [19], [20]), conclusive evidence has been lacking due to the methodological limitations of behavioral studies. Focusing on the question of whether payoff is necessarily weighted by probability when making a risky choice, the present study investigated the differences between preferential choices and trade-off instruction choices from the perspective of functional integration. We found that the regions obtained by conjunction analyses could be further divided into two networks within each task condition. Each of the networks consisted of regions that previous studies had reported as being associated with probability representation or payoff representation. Furthermore, by investigating the functional connectivity of the probability or payoff networks, we found that the functional connectivity between the two networks was stronger during the trade-off instruction choice task. We found that the response pattern that would be expected for a weighting algorithm was detected in the trade-off instruction choice task but was unnecessary in the preferential choice task. This provides neural evidence that preferential choice is not based on an expectation computation, as has been postulated by expectation theories.

Decision making is a complex cognitive process that involves multiple brain regions, including the prefrontal and parietal lobes and the striatum [33], [66], [67]. These brain regions do not act in isolation, but rather must work together as a system [22]. Therefore, one challenge for understanding the neurobiological mechanisms of decision making is to determine how the components of large-scale distributed neural systems are coupled together during decision making [22], [25]. In the present study, we found that the regions participating in the risky decision making tasks could be categorized into two networks that were separately implicated in probability representation and payoff representation, during both the preferential choice task and the trade-off instruction choice task.

In the so-called probability network, the bilateral PFC and the PPC were activated in both tasks. These regions have consistently been identified in previous studies on risky decision making and have been implicated in the representation and processing of probability [28]–[30], [34], [35]. Furthermore, we found that the bilateral anterior insula was also included in the probability network in both tasks. The activity of the insula, which has been traditionally implicated in interoception perception and negative valence affective processes [68], [69], has a broad range of risk-related decision-making characteristics as well. Data on its specific role in decision-making under risk are inconsistent in separate studies. Some studies have found that the activity of the insula is related to uncertainty [36], [62] or risk per se [63] and that it may signal the possibility of aversive outcomes [30], [70]–[72]. Other studies have suggested that the insula is also sensitive to reward magnitude [73], [74]. However, a recently published quantitative meta-analyses of functional magnetic resonance imaging experiments on risk processing, in which a range of experiments and paradigms was analyzed, found that risk, whether decision risk or anticipation risk, is consistently represented in the anterior insula [75]. Based on the connectivity matrix and the results of the clustering analysis in which the anterior insula showed strong functional connectivity to the fronto-parietal regions that have been previously implicated in the representation of probability, we speculate that the insula may also be implicated in the representation of probability by signaling the possibility of outcomes.

In the so-called payoff network, the striatum is the structure most consistently reported to be involved in the influence of reward magnitude/payoff on the neural substrate. Although many neuroimaging studies of reward processes have focused on ventral striatal activation, a growing body of literature from animal and human studies [40], [55] suggests that the dorsal striatum is also involved in motivated behaviors. In a study which directly measured the effects of reward or punishment magnitude in the striatum by scanning participants while they anticipated rewards and punishments that varied in amounts, researchers found that ventral striatum activity was associated with the anticipation of larger rewards, whereas the dorsal striatum was activated when both rewards and punishments of larger magnitude were anticipated [76]. The role of the thalamus in response to reward anticipation has been studied less extensively. However, some studies have associated reward effects with neural responses in the thalamus [73], [77]. For example, the anticipation of increasing rewards and punishments has been found to activate the anterior thalamus [76]. The finding of strong functional connectivity within the payoff network is consistent with evidence that the striatum has extensive anatomical interaction with the thalamus [78], and this is also consistent with the involvement of both regions in decision-making [79]. The pallidum receives information from the striatum and sends fibers to the thalamus [33]. Because the latter two regions are implicated in reward representation, the pallidum may possibly participate in transferring information about payoffs.

The finding that these regions with similar functions can be grouped into distinct networks suggests that two different networks responsible for exchanging information about probabilities and payoffs exist in both preferential and trade-off instruction choice tasks. Therefore, if the strength of functional connectivity between the two networks in the preferential choice task is different from the strength in the trade-off instruction choice task, there may be differences in the extent of cooperation of the processing of probability and payoff occurring between tasks. As we hypothesized, the functional connectivity between the payoff network and regions within the probability network (i.e., the bilateral IPL and the left lateral PFC) were significantly stronger during the trade-off instruction choice task than during the preferential choice task. The bilateral IPL, a part of the PPC which has been reported to be critical for representations of probability [28], [29], may participate in inexact calculation and numerical estimation [27]. The canonical perspective on decision-making under risk posits that a complex value estimation (e.g., working with weighted functions) is involved in choice under risk. The stronger connectivity between the bilateral IPL in the payoff network during the trade-off instruction choice task provides evidence for our hypothesis that the preferential choice is unlike the trade-off instruction choice, which requires a strong integration between the information on probabilities and payoffs to make a choice under risk.

Further support for this view is provided by the significantly increased functional connectivity between the left dorsolateral PFC and the payoff network during the trade-off instruction choice task compared to the connectivity found during the preferential choice task. In the present study, two regions in the left dorsolateral PFC showed stronger connectivity with the payoff network during the trade-off instruction choice task. One of the regions (the left pIFS) was located in clusters reported in previous studies [30], [31], [36]. Its activity was stronger when participants selected options with lower probability in the context of invariant reward magnitudes [30]. In addition, its involvement in decision-making under risk may be related to its role in cognitive control [31] and in coding less predictable choices [80], [81]. The other frontal region (the aPFC), located in the anterior part of the dorsolateral PFC, has been reported to exhibit activity in connection with working memory, relational integration, and problem solving [32], [82]–[84]. In terms of decision making, this region tends to be the one that is the most involved in manipulating decision-relevant information on-line, in conscious deliberation during decisions and in making decisions under uncertain circumstances in which no objectively correct answer can be identified [33]. In particular, during decision making that is simple and is well structured in terms of goals and options, as in our case, the left aPFC may play a privileged role in the decision process [33]. In the payoff network, the striatum is the main output structure, receiving afferents from specific cortical areas to project them to the thalamus and the brainstem for additional propagation via the pallidum back to the cortex [85]. Evidence from previous studies confirms the central role of the dorsolateral PFC input to the striatum in the modulation of goal-directed behavior [86]. The anatomy of the integrative striatal pathways enables the transfer of a reward representation from the ventral striatum into an optimal behavior output via the connections of the dorsal striatum to the dorsolateral PFC [87]. Based on these findings, a credible explanation for the increased connectivity between the left dorsolateral PFC and the regions related to payoffs during the trade-off instruction task could be that the trade-off instruction choice task involves more cognitive operations and deliberate actions than the preferential choice task. Specifically, considering the existence of the left dorsolateral PFC in the probability-related network, the increased connectivity further suggests that the integration of probability and payoff to achieve a risky decision seems to be required more for a trade-off instruction choice task than for a preferential choice task.

We found that the functional connectivity between networks in the gain domain was not significantly different from those in the loss domain, indicating that individuals do not employ different weighting strategies in different domains. In other words, it seems unlikely that individuals integrate probabilities and payoffs to achieve a risky decision (i.e., use a compensatory rule) in the gain domain while not integrating probabilities and payoffs to make decisions (i.e., use a non-compensatory rule) in the loss domain, or vice versa. Such a finding suggests that our results, which indicate that payoffs are not necessarily weighted by their respective probabilities when making risky choice, are independent of domain.

In this study, we observed increased functional connectivity between the payoff network and some regions in the probability network during the trade-off instruction choice task than during the preferential choice task. We noticed a longer RT in the tradeoff instruction choice reported in our previous study using the same data but focusing on a different topic [26]. Thus, the increased functional connectivity during the tradeoff instruction choice task can be argued to be caused by the trade-off instruction choice task being difficult than the preferential choice task. If longer RT suggests a more difficult task, a stronger activation in brain regions during the trade-off instruction choice task than during the preferential choice task should be observed. However, by analyzing the differences in activation extent in the seed regions of functional connectivity, we found a stronger activation in the preferential choice than in the trade-off instruction choice. Therefore, this finding suggests that the increased functional connectivities during the trade-off instruction choice task may be not due to the possibility of the compensatory decision-making task being more difficult than the non-compensatory task. This is our tentative explanation that requires further validation.

In this study, we were interested in the differences in functional connectivity between regions involved in probability representation and in payoff representation. Both types of information are processed in the preferential choice task and in the trade-off instruction task according to our task requirements. Therefore, we only focused on the regions that were activated in both the preferential choice task and in the trade-off instruction task. This ruled out some regions in our analyses, such as the bilateral nucleus accumbens, which was significantly activated in the preferential choice task but not in the trade-off choice task. We categorized the regions activated in our study into the probability or the payoff network based on their interregional connectivity characteristics. This rationale is based on the notion that cognition results from the interactions between distributed brain regions operating as large-scale networks. This notion emphasizes the conjoint function of brain areas working together as large-scale networks rather than a simplistic mapping of cognitive constructs onto individual brain areas [88]. It is worth noticing that probability processing is a complex cognition process, which may include mathematical calculation, attention, cognitive control and other factors. Payoff information processing is a similarly complex process, including reward, concomitant emotion and salience processing. Along this line, both probability and payoff processing should result from interactions of distributed brain areas operating in large-scale networks. In the current study, we found strong functional connectivity among the bilateral PPC, lateral PFC and insula in the two different risky decision-making tasks. Although individual regions are involved different functions, such as mathematical calculation [27], cognitive control [31] and signaling risk [36], [62], [63], in our tasks, they worked together as a network and thus may cooperate to accomplish one job. This job is likely the processing of information about probability in our task contexts. Therefore, we termed the network consisting of the bilateral PPC, lateral PFC and insula the probability network. Similarly, we found a strong functional connectivity between the bilateral dorsal and ventral striatum and the thalamus in both of the risky decision-making tasks. Combining functions of the individual one of these regions, such as reward [40]–[42], [73], [77] and salience processing [89], with the job which needed these region worked together as a network to accomplish in our task contexts, we think that these regions may cooperate in processing payoff information. Therefore, we termed the network constituted by the bilateral dorsal and ventral striatum and the thalamus the payoff network. However, it must be noted that the results from this study cannot be used to conclusively ascertain whether the activities of these regions working together as a network are modulated by the magnitude of the probabilities or payoffs. Future studies that directly link the activities of these regions and the magnitude of the probabilities or payoffs should be performed. Additionally, because this is a preliminary study, our sample size is relatively small. Studies with larger sample sizes are needed to validate our findings.

In summary, using functional connectivity analyses, we found that the connectivity between regions associated with probability and the magnitude of reward were stronger during the trade-off instruction choice task than during the preferential choice task. This increased functional integration during the trade-off instruction choice task supports our hypothesis that payoffs are not necessarily weighted by their probability when making a risky choice. Instead, preferential choice may possibly be guided by a non-compensatory process, in which decision making depends on one single dimension. This type of neural exploration utilizes a functional integration perspective and indicates a new research direction. This approach may be useful for the investigation of the expectation principle and may provide a new perspective for revealing the neural mechanism of decision making.
